# Effects of the Best Possible Self intervention: A systematic review and meta-analysis

**DOI:** 10.1371/journal.pone.0222386

**Published:** 2019-09-23

**Authors:** Alba Carrillo, María Rubio-Aparicio, Guadalupe Molinari, Ángel Enrique, Julio Sánchez-Meca, Rosa M. Baños

**Affiliations:** 1 Department of Personality, Assessment and Psychological Treatments, University of Valencia, Valencia, Spain; 2 Department of Health Psychology, University of Alicante, Alicante, Spain; 3 Department of Basic, Clinical and Biological Psychology, University Jaume I, Castellón, Spain; 4 CIBER Fisiopatología Obesidad y Nutrición (CIBEROBN), Instituto Carlos III, Madrid, Spain; 5 School of Psychology, University of Dublin Trinity College, Dublin, Ireland; 6 Department of Basic Psychology and Methodology, University of Murcia, Murcia, Spain; Universitat Wien, AUSTRIA

## Abstract

The Best Possible Self (BPS) exercise promotes a positive view of oneself in the best possible future, after working hard towards it. Since the first work that attempted to examine the benefits of this intervention in 2001, studies on the BPS have grown exponentially and, currently, this is one of the most widely used Positive Psychology Interventions. However, little is yet known about its overall effectiveness in increasing wellbeing outcomes. Thus, the aim of this meta-analysis is to shed light on this question. A systematic literature search was conducted, and 29 studies (in 26 articles) met the inclusion criteria of empirically testing the intervention and comparing it to a control condition. In addition, BPS was compared to gratitude interventions in some of the included studies. A total of 2,909 participants were involved in the analyses. The outcome measures were wellbeing, optimism, depressive symptoms, and positive and negative affect. Results showed that the BPS is an effective intervention to improve wellbeing (*d*_+_ = .325), optimism (*d*_+_ = .334) and positive affect (*d*_+_ = .511) comparing to controls. Small effect sizes were obtained for negative affect and depressive symptoms. Moderator analyses did not show statistically significant results for wellbeing, except for a trend towards significance in the age of the participants (years) and the magnitude of the intervention (total minutes of practice). In addition, the BPS was found to be more beneficial for positive and negative affect than gratitude interventions (*d*_+_ = .326 and *d*_+_ = .485, respectively). These results indicate that the BPS can be considered a valuable Positive Psychology Intervention to improve clients’ wellbeing, and it seems that it might be more effective for older participants and with shorter practices (measured as total minutes of practice).

## Introduction

Since the beginning of the Positive Psychology movement, research on positive functioning and wellbeing has grown exponentially [1]. Many efforts have been made to develop and validate different Positive Psychology Interventions (PPIs), defined as interventions or intentional activities whose aim is to cultivate positive feelings, cognitions, and behaviors [2]. Several meta-analyses have shown that these interventions are, in general, effective in increasing wellbeing levels and decreasing depressive symptoms [2,3]. Specifically, the latest meta-analysis of the effectiveness of PPIs [3] revealed small to moderate effect sizes for wellbeing (*d* = .20 for psychological wellbeing and *d* = .34 for subjective wellbeing) [4]. These reviews provided very relevant data about the effectiveness of PPIs, but they included a wide range of interventions. Even though PPIs share the same aim, they are quite heterogeneous in their specific target (e.g. interventions that promote optimism, gratitude, or social connectedness), form (e.g. writing a gratitude letter or savoring the moment), and dosage (e.g. one single session or a 1-month program), and they are delivered through different procedures (e.g. individually or in groups, face-to-face or online). Therefore, their analyses and conclusions are rather generic and do not provide specific information about the effectiveness of a particular PPI. Thus, more precise reviews are needed to complement their results.

One widely used PPI is the “Best Possible Self” (BPS) intervention, which consists of writing about one’s best possible self in the future after everything has gone as well as it possibly could. The first study to use this paradigm compared its effectiveness to a disclosive writing condition about a traumatic event [5]. Results indicated that the BPS produced significant improvements in wellbeing at posttest, and it was rated as less upsetting than a trauma-writing exercise. Since the publication of this work, many studies have been carried out on the BPS using different approaches and delivery methods [6,7].

Due to the large number of studies on the BPS, some reviews have been performed. A recent meta-analysis of the effectiveness of interventions to increase optimism showed a moderate effect size (*g* = .41) for experimental conditions compared to controls on optimism levels [4,7]. This review included any intervention designed to improve optimism levels, comprising not only BPS studies but also other interventions. Moderator analyses showed that BPS was more effective (*g* = .64) than other interventions (*g* = .28) in increasing optimism levels. However, this review only addressed optimism as the outcome variable, and, therefore, it exclusively analyzed BPS studies that measured optimism (k = 10). Recently, a qualitative review of BPS interventions [6] concluded that the BPS is a recommended intervention to improve wellbeing, which is flexible in its delivery (i.e. online or face-to-face) and implementation (e.g. written or spoken). However, neither quantitative analyses of the effectiveness of the interventions nor evaluations of the quality of the studies were performed. Therefore, almost two decades after the first study on the BPS, little is known about the overall effectiveness of this intervention on wellbeing. A quantitative and systematic approach is needed to shed light on the effectiveness of this intervention and to analyze potential moderators that can influence its effectiveness.

Consequently, the aim of the present study is to conduct a meta-analysis of the effectiveness of the BPS intervention on wellbeing compared to controls. Potential moderators will be examined, as well as the methodological quality of the studies. Additionally, if there are enough studies with other experimental conditions equivalent to the BPS (i.e. another type of PPI), further comparisons of their effectiveness will be carried out.

## Method

This meta-analysis was carried out following the Preferred Reporting Items for Systematic Reviews and Meta-analysis (PRISMA) guidelines [8]. S1 Checklist presents the PRISMA 2009 Checklist.

### Eligibility criteria

The inclusion criteria were:

1. Empirical test of the effects of the BPS intervention. A BPS intervention was defined as an exercise in which participants write about the best version of themselves in the future after everything has gone as well as possible [5,9,10]. Studies that included this intervention as part of a multi-component intervention but did not analyze the effects of the BPS separately, were excluded.
2. A minimum of two groups, one BPS condition and one control condition (whether active or waiting list). The active control conditions were defined as active neutral exercises not considered PPIs, such as writing about one’s daily activities.
3. At least one measure of wellbeing (e.g. wellbeing, satisfaction with life, positive affect, happiness), optimism, or depression, and two time points (before–pretest, and after the intervention–posttest).
4. Enough statistical data to perform the calculations of the standardized effect sizes (means and standards deviations of the different groups at pretest and posttest). If necessary, authors would be contacted to provide missing information.
5. Study written in English or Spanish.

### Search strategy

A systematic literature search was carried out in the PsychInfo, ISI Web of Science, Cochrane, Scopus, and PubMed databases, including all the works published until November 2017 (when the search was conducted). In addition, this search was carried out in the databases of the main journals that commonly published works on PPIs: Journal of Positive Psychology, Journal of Happiness Studies, and Social Indicators Research. The terms used in the search were the two names used for the intervention in the initial study and later published studies: “Best Possible Self” and “Best Possible Selves”. Furthermore, systematic reviews of PPIs [2,3,6,7] and the references from the retrieved studies were revised, and experts in the field were consulted. Finally, a cited reference search for the initial work on the BPS by King [5] was carried out in the ISI Web of Science database, looking for all works that cited this original paper.

### Outcome measures

In this meta-analysis, several outcome measures were included: wellbeing (which included measures of wellbeing, positive and negative affect, life satisfaction, or happiness), optimism (because the BPS intervention is a future-oriented positive activity that promotes a positive outlook on the future), and depressive symptoms.

For wellbeing, the most frequent scales used were the Positive and Negative Affect Schedule (PANAS) [11], the Satisfaction With Life Scale (SWLS) [12], the Brief Multidimensional Students’ Life Satisfaction Scale (BMSLSS) [13], the Warwick-Edinburgh Mental Well-being Scale (WEMWBS) [14], and the Subjective Happiness Scale (SHS) [15]. Optimism was mainly measured with the Future Expectancies Scale (FEX) [16], the Life Orientation Test-Revised (LOT-R) [17], the Subjective Probability Task (SPT) [18], and the Attributional Style Questionnaire (ASQ) [19]. Finally, depression was measured by the Centre of Epidemiological Studies Depression Scale (CES-D) [20], the State-Trait-Anxiety-Depression Inventory (STADI) subscales of state euthymia (inverted), and state dysthymia [21], and the Beck Depression Inventory (BDI-II) [22].

### Study selection criteria

Selection of studies was carried out independently by two reviewers (AC and GM). After duplicates were removed, the studies were screened by title and abstract. When at least one of the coders selected a study as potentially eligible, this study passed to the second phase. In this phase, the selected studies went through a full-text analysis by both reviewers. Inconsistencies between the coders were resolved by consensus.

Kappa coefficients (for the categorical variables) and intra-class correlations (for the continuous variables) were calculated to check the reliability of the coding process.

### Coding of moderator variables

Extracted data were:

1. Delivery method of the intervention: information was collected about whether the exercise was applied individually (i.e. only one participant at a time) or in groups (i.e. more than one participant at a time in the same room), and face-to-face (e.g. if participants attended laboratory sessions or the exercise was applied in a room at the University) or online (i.e. if participants received the instructions through a webpage and did not physically attend a practice session).
2. Contextual aspects: whether participants received compensation for participating (in the form of money or University credits) as a reflection of the intrinsic motivation of participants.
3. Components of the intervention. The BPS is a PPI that requires participants to envision themselves in the future. However, not all studies have included explicit instructions to visualize the written content or a specific period of time to perform the visualization (e.g. 5 minutes). Therefore, the “imagery component” variable was coded as present if the study described an explicit method to implement this visualization in the practice of the exercise.
4. Duration of the intervention. Interventions in BPS studies have had different durations (e.g. one day or one month) and different practice intensities (e.g. daily practice or once a week). Consequently, three variables were coded in this area as potential moderators: length, intensity, and magnitude. Length refers to the total number of days that participants practiced the exercise (e.g. 7 days). Intensity refers to the number of minutes of practice *per week* (e.g. 20 minutes per week). Finally, magnitude refers to the *total* number of minutes of practice. When this information was not directly provided in the studies, it was calculated by the authors of this paper.
5. Population. Lately, some authors have highlighted the relevance of personal characteristics of participants who practice PPIs, pointing out the need to examine sociodemographic variables when assessing the efficacy of these activities [23]. Previous studies have shown that variables such as age, sex or country of origin play an important role on the effects produced by these activities (e.g. [24–27]). In this meta-analysis, the following data were collected: country of the study (later grouped by continent), target population (community or undergraduate students), age, percentage of women, and group sizes. The sample size in the studies was included as they might produce differences in the results (i.e. larger sample sizes could present greater heterogeneity among the participants in the sample).

### Quality of the studies

The specific characteristics of each meta-analysis lead to elaborate precise items for assessing methodological quality of primary studies. In this case, the methodological quality of the included studies was assessed with a 9-item scale [28], based on items usually included in many of the quality scales and checklists proposed in the literature. In particular, the quality criteria used were mainly based on the PEDRO scale [29] and on the risk of bias items from the Cochrane Collaboration [30].

Each criterion was rated as 0 when the criterion was not met (or not reported), or 1 when the criterion was met. The following criteria were included: (1) randomized assignment of participants; (2) baseline comparability between experimental and control conditions (i.e., if groups were matched on pretest measures or whether there were no statistically significant differences between the groups at pretest on relevant variables); (3) baseline comparability between dropouts and completers (if there were no dropouts, this item was also coded as 1); (4) type of control group (active group coded as 1, and waiting list coded as 0); (5) concealment of assessors of the participants’ assigned condition; (6) standardized scales used to assess the outcome measures; (7) attrition rate ≤ 10%; (8) intention-to-treat analyses (if there were no dropouts, this item was also coded as 1); and (9) reporting bias (if all measures described in the method section were reported in the results section).

### Computation of effect sizes

The effect size index was the standardized mean difference between the change scores of the BPS and control groups. This index, although scarcely used in practice, has the advantage of controlling for pretest differences between the groups, as well as for maturation, history, or testing effects from pretest to posttest [31,32]. For each study, this index was calculated by subtracting the mean pretest-posttest difference of the control group (${\overset{-}{y}}_{pre,C}$ and ${\overset{-}{y}}_{pos,C}$) from the mean pretest-posttest difference of the experimental group (${\overset{-}{y}}_{pre,T}$ and ${\overset{-}{y}}_{pos,T}$), and dividing this difference by the pooled standard deviation of both groups on the pretest (*S*_*pre*_), with c(m) being a correction factor for small sample sizes:

$$d=c(m)[\frac{\left({\overset{-}{y}}_{pre,T}-{\overset{-}{y}}_{pos,T})-({\overset{-}{y}}_{pre,C}-{\overset{-}{y}}_{pos,C}\right)}{S_{pre}}]$$ (1)

In general, the *d* index was calculated to compare the BPS and control groups. However, we found that 7 of the included studies contained a gratitude group *in addition* to a control group. That is, these studies included an extra group that practiced a PPI designed to increase or promote feelings of gratitude, such as writing down things that went well during the day or writing a letter of gratitude [33]. In these cases, the *d* index was also applied to compare the BPS and gratitude groups (independently of the *d* index that compared the BPS and control groups). Positive *d* values indicated a better result for BPS than for the control and gratitude groups.

In each study, a *d* index was calculated for each of the three different types of outcomes (wellbeing, optimism, and depression). The calculations of *d* indices for wellbeing encompassed measures of satisfaction with life, happiness, wellbeing, positive affect, and negative affect (this measure was inverted for the calculus of the wellbeing *d* index). Optimism was composed of measures of optimism and positive future expectancies. Regarding depression, only instruments that explicitly addressed depressive symptoms were included. Additionally, due to the large number of studies that applied the PANAS scale [11], two additional meta-analyses were carried out for the positive and negative affect outcomes measured with this instrument. Thus, two additional *d* indices were calculated in the studies that included the PANAS scale, one for positive affect and another for negative affect. Hence, the number of separate meta-analyses was increased to five, analyzing the effectiveness of the intervention for wellbeing, optimism, depression, positive affect, and negative affect.

When a study applied several measures of the same construct (e.g., two different scales of optimism), a *d* index was calculated for each measure. Then, in order to avoid dependence problems, they were averaged to represent the specific study, with a *d* value only for that type of outcome (optimism in the example). Separate meta-analyses were conducted for each type of outcome, and the individual studies did not necessarily have to include measures of all of them. For example, there were studies that only reported measures for wellbeing and optimism, but not for depression, and these studies contributed only to the corresponding meta-analyses.

### Statistical analyses

Separate meta-analyses were carried out with the effect sizes for each of the five outcomes and for the comparison of the BPS with the control and gratitude groups.

In order to address the variability exhibited by the effect sizes, a random-effects model was assumed [34,35]. This model involves weighting each effect size by its inverse variance, defined as the sum of the within-study and between-studies variances. The between-studies variance was estimated using restricted maximum likelihood.

The interpretation of the clinical significance of the mean effect sizes obtained in this work was assessed by comparing them with the 25%, 50%, and 75% percentiles of the distribution of effect sizes obtained in a methodological review of 50 meta-analyses within the field of the effectiveness of clinical psychological interventions [31], which was considered as a representative means of interpreting the effect sizes of psychological interventions.

Several analyses were carried out in order to test whether publication bias could be a threat to the validity of the meta-analytic results. In particular, the Egger test was applied, and funnel plots were constructed with the trim-and-fill method [36]. The Egger test consists of constructing an unweighted simple regression, with the effect size as the dependent variable and the standard error of each effect size as the independent variable. A statistically non-significant result of the *t*-test for the hypothesis of an intercept equal to zero permits to discard publication bias.

Heterogeneity among the effect sizes was assessed with the *Q* statistic and the *I*^*2*^ index. *I*^*2*^ values of approximately 25%, 50%, and 75% can be considered to reflect low, moderate, and large heterogeneity, respectively [37].

Assuming a mixed-effects model, the influence of moderator variables on the effect sizes was calculated through ANOVAs and meta-regressions for the categorical and continuous variables, respectively [38,39]. The improved method proposed by Knapp and Hartung [40] was applied to test the statistical significance of each moderator variable. The *F* statistic makes it possible to test the statistical association between a moderator variable and the effect sizes, and the *Q*_E_ and *Q*_W_ statistics enable us to examine the model misspecification for the continuous and categorical moderators, respectively. Statistically significant results for the *Q*E and *Q*W indicate whether the ANOVAs and simple meta-regressions are misspecified, that is, whether other moderator variables could also affect the effect size variability. In addition, an estimate of the proportion of variance accounted for by the moderator variable was calculated by means of $R^{2}=1-{\hat{\tau }}_{Res}^{2}/{\hat{\tau }}_{Total}^{2}$, with ${\hat{\tau }}_{Res}^{2}$ and ${\hat{\tau }}_{Total}^{2}$ being the residual and total heterogeneity variance estimates, respectively [41]. Following the recommendations of Aguinis, Gottfredson, and Wright [42] and Viechtbauer et al. [39], moderator analyses were applied only for outcome measures with at least 20 studies (i.e., wellbeing).

The statistical analyses were carried out with the *metafor* package in *R* [43].

## Results

### Coding reliability

To check the reliability of the coding process of the study characteristics, all studies were doubly coded by two independent coders (AC and GM). The results were highly satisfactory overall, with kappa coefficients ranging between .684 and 1.0 (M = .920) for qualitative characteristics, and intra-class correlations between .958 and 1.0 (M = .994) for the continuous variables. The inconsistencies between the coders were resolved by consensus.

### Descriptive characteristics of the studies

**Fig 1:**
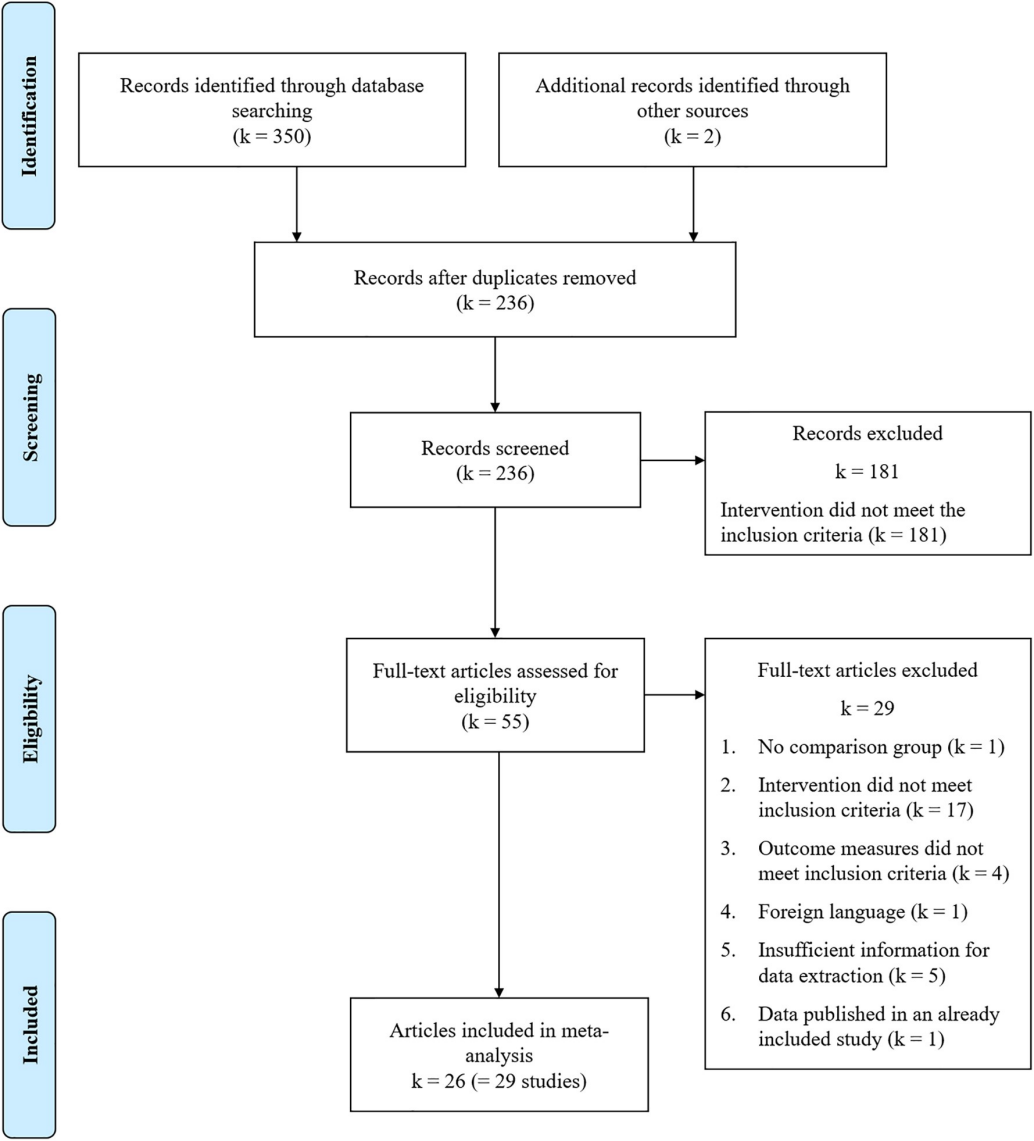
Flow diagram.

The selection process is illustrated in Fig 1. First, 350 titles were retrieved from the databases, and 2 additional titles were retrieved by searching reference lists and consulting experts. After duplicates were removed, 236 records were screened, and 181 of them were excluded after reading the abstracts. Finally, 55 articles were selected as potentially eligible studies, of which 29 did not meet the inclusion criteria.

Main characteristics of the studies can be found in Table 1. One article included two studies [44], and two articles included BPS and control groups delivered through different methods, either writing or talking [45], or online or face-to-face [46]. These comparisons were treated as independent studies. One of the included studies was an unpublished dissertation [47], and one of them was a conference proceeding [48].

**Table 1:** Characteristics of the studies that examined the effects of the BPS intervention.

Study	Delivery method, Contextual aspects, Components	Length (days), Intensity (minutes/week), Magnitude (total minutes)	N analyzed	Population, country, age (M, SD) % women	Outcome measures
Boehm et al. (2011) [26]	Individual + online, Compensation, NO visualization	Days = 42, Minutes/week = 10, Total minutes = 60	BPS = 72 Control (A) = 70 Gratitude = 71 *Total = 213*	Community, USA, 35.62 (11.36), 52.7%	LS: SWLS
Boselie et al. (2014) [60]	Individual + face-to-face, Compensation, Visualization	Days = 1, Minutes/week = 20, Total minutes = 20	BPS = 38 Control (A) = 36 *Total = 74*	Undergraduate, Netherlands, 21.90 (2.29), 78.4%	PA, NA: PANAS Opt: FEX
Boselie et al. (2016)a [44]	Individual + face-to-face, Compensation, Visualization	Days = 1, Minutes/week = 20, Total minutes = 20	BPS = 41 Control (A) = 40 *Total = 81*	Undergraduate, Netherlands, 21.35 (4.28), 79%	PA, NA: PANAS Opt: FEX
Boselie et al. (2016)b [44]	Individual + face-to-face, Compensation, Visualization	Days = 1, Minutes/week = 20, Total minutes = 20	BPS = 32 Control (A) = 29 *Total = 61*	Undergraduate, Netherlands, 21.84 (2.22), 73.8%	PA, NA: PANAS Opt: FEX
Boselie et al. (2017) [62]	Individual + face-to-face, Compensation, Visualization	Days = 1, Minutes/week = 20, Total minutes = 20	BPS = 31 Control (A) = 30 *Total = 61*	Undergraduate, Netherlands, 21.48 (2.47), 90.2%	PA, NA: PANAS Opt: FEX
Enrique et al. (2017) [58]	Individual + face-to-face, NO compensation, Visualization	Days = 30, Minutes/week = 35, Total minutes = 170	BPS = 38 Control (A) = 40 *Total = 78*	Under+Comm, Spain, 23.80 (3.85), 65.4%	PA, NA: PANAS Opt: LOT, SPT Dep: BDI-II
Geschwind et al. (2015) [59]	Individual + face-to-face, Compensation, Visualization	Days = 1, Minutes/week = 20, Total minutes = 20	BPS = 25 Control (A) = 25 *Total = 50*	Under+Comm, Belgium, 20.32 (1.97), 100%	PA: mDES
Hanssen et al. (2013) [16]	Individual + face-to-face, Compensation, Visualization	Days = 1, Minutes/week = 20, Total minutes = 20	BPS = 40 Control (A) = 39 *Total = 79*	Undergraduate, Netherlands, 22.59 (2.86), 81%	PA, VAS* Opt: FEX
Harrist et al. (2007)a [45]	Individual + face-to-face, Compensation, NO visualization.	Days = 1, Minutes/week = 20, Total minutes = 20	BPS = 19 Control (A) = 20 *Total = 39*	Undergraduate, USA, 21 (?), 61.5%	PA, NA: Diener & Emmons, 1984
Harrist et al. (2007)b [45]	Individual + face-to-face, Compensation, NO visualization	Days = 1, Minutes/week = 20, Total minutes = 20	BPS = 18 Control (A) = 18 *Total = 36*	Undergraduate, USA, 21 (?), 72.2%	PA, NA: Diener & Emmons, 1984
King (2001) [5]	Individual + face-to-face, Compensation, NO visualization	Days = 1, Minutes/week = 20, Total minutes = 20	BPS = 19 Control (A) = 16 *Total = 35*	Undergraduate, USA, 21.04 (3.15), 83.1%	PA: Diener & Emmons, 1984
Layous et al. (2013)a [46]	Group + face-to-face, Compensation, NO visualization	Days = 28, Minutes/week = 15, Total minutes = 60	BPS = 50 Control (A) = 23 *Total = 73*	Undergraduate, USA, 19.10 (1.77), 71.8%	PA: Diener & Emmons, 1984
Layous et al. (2013)b [46]	Individual + online, Compensation, NO visualization	Days = 28, Minutes/week = 15, Total minutes = 60	BPS = 32 Control (A) = 16 *Total = 48*	Undergraduate, USA, 19.10 (1.77), 71.8%	PA: Diener & Emmons, 1984
Liau et al. (2016) [52]	Individual + face-to-face, NO compensation, NO visualization	Days = 30, Minutes/week = 20, Total minutes = 40	BPS = 81 Control (A) = 81 *Total = 162*	Undergraduate, Singapore, 17.83 (1.12), 73.8%	PA, NA: PANAS LS: BMSLSS Dep: CES-D Opt: LOT-R
Lyubomirsky et al. (2011) [61]	Individual + online, Compensation, NO visualization	Days = 56, Minutes/week = 15, Total minutes = 120	BPS = 112 Control (A) = 110 Gratitude = 108 *Total = 330*	Undergraduate, USA, 19.66 (2.91), 71.2%	PA, NA: Barret & Russell, 1988 LS: SWLS H: SHS
Maddalena et al. (2014) [56]	Individual + face-to-face, Compensation, NO visualization	Days = 30, Minutes/week = 20, Total minutes = 60	BPS = 23 Control (A) = 9 *Total = 32*	Undergraduate, USA, 20.70 (3.80), 55.8%	NA: POMS Opt: LOT LS: SWSL
Manthey et al. (2016) [63]	Individual + online, NO compensation, NO visualization	Days = 56, Minutes/week = ?, Total minutes = ?	BPS = 135 Control (A) = 150 Gratitude = 150 *Total = 435*	Under+Comm, Germany, 33.70 (9.60), 84.1%	PA, NA: SPANE LS: SWLS Dep: STADI
Meevissen et al. (2011) [10]	Individual + face-to-face, Compensation, Visualization	Days = 14, Minutes/week = 35, Total minutes = 90	BPS = 28 Control (A) = 23 *Total = 51*	Undergraduate, Netherlands, 23.50 (6.39), 92.6%	PA, NA: PANAS Opt: LOT, SPT
Meevissen et al. (2012) [48]	Individual + face-to-face, Compensation, Visualization	Days = 1, Minutes/week = 20, Total minutes = 20	BPS = 37 Control (A) = 35 *Total = 72*	Undergraduate, Netherlands, 21.30 (2.10), 100%	PA, NA: BMIS Opt: FEX
Ng (2016) [64]	Individual + face-to-face, Compensation, NO visualization	Days = 21, Minutes/week = ?, Total minutes = ?	BPS = 118 Control (A) = 98 *Total = 216*	Undergraduate, Singapore, 28 (?), 63.4%	H: SHS
Odou & Vella-Brodrick (2013) [49]	Individual + online, NO compensation, Visualization	Days = 7, Minutes/week = ?, Total minutes = ?	BPS = 73 Control (WL) = 67 Gratitude = 70 *Total = 210*	Community, Australia, 34 (13.99), 74.8%	PA, NA: PANAS WB: WEMWBS
Peters et al. (2010) [54]	Group + face-to-face, NO compensation, Visualization	Days = 1, Minutes/week = 20, Total minutes = 20	BPS = 44 Control (A) = 38 *Total = 82*	Undergraduate, Sweden, 29.60 (?), 62.2%	PA, NA: PANAS Opt: SPT
Peters et al. (2013) [55]	Individual + face-to-face, Compensation, Visualization	Days = 7, Minutes/week = 55, Total minutes = 55	BPS = 28 Control (A) = 28 Gratitude = 26 *Total = 82*	Undergraduate, Netherlands, 22.80 (?), 84.1%	LS: SWLS Opt: LOT-R, ASQ
Peters et al. (2016) [50]	Individual + face-to-face Compensation, Visualization	Days = 1, Minutes/week = 20, Total minutes = 20	BPS = 28 Control (A) = 28 *Total = 53*	Undergraduate, Germany, 23.50 (3.30), 57.14%	PA, NA: PANAS Opt: LOT, SPT
Renner et al. (2014) [51]	Individual + face-to-face, Compensation, Visualization	Days = 1, Minutes/week = 20, Total minutes = 20	BPS = 20 Control (A) = 20 *Total = 40*	Undergraduate, Netherlands, 22.10 (?), 80%	PA, NA: PANAS
Sheldon et al. (2006) [53]	Group + face-to-face, NO compensation, NO visualization	Days = 28, Minutes/week = ?, Total minutes = ?	BPS = 23 Control (A) = 23 Gratitude = 21 *Total = 67*	Undergraduate, USA, ? (?), 74.6%	PA, NA: PANAS
Summerfield (2015) [47]	Individual + online, Compensation, Visualization	Days = 5, Minutes/week = 75, Total minutes = 75	BPS = 15 Control (A) = 15 Gratitude = 15 *Total = 45*	Under+Comm, UK, ? (?), 73.3%	PA, NA: PANAS LS: SWLS
Troop et al. [65]	Group + face-to-face, NO compensation, NO visualization	Days = 14, Minutes/week = 22.5, Total minutes = 45	BPS = 23 Control (A) = 23 *Total = 46*	Undergraduate, UK, 25.8 (9.3), 67.39%	PA: TPAS
Yogo et al. (2008) [66]	Individual + face-to-face, NO compensation, NO visualization	Days = 1, Minutes/week = 20, Total minutes = 20	BPS = 27 Control (A) = 28 *Total = 55*	Undergraduate, Japan, ? (?), 71.15%	NA: MMS

Abbreviations (in alphabetical order): A = active; ASQ = Attributional style questionnaire; BDI-II = Beck depression inventory–II; BMIS = Brief mood introspection scale; BMSLSS = Brief multidimensional students’ life satisfaction scale; BPS = Best Possible Self; CES-D = Center for epidemiologic studies depression scale; Dep = Depressive symptoms; FEX = questionnaire for future expectations; H = Happiness; LOT / LOT-R = Life orientation test / revised; LS = Life Satisfaction; M = Mean; mDES = Modified differential emotions scale; MMS = Multiple mental states; NA = Negative Affect; Opt = Optimism; PA = Positive Affect; PANAS = Positive and negative affect schedule; POMS = Profile of mood states; SD = standard deviation; SHS = Subjective happiness scale; SPANE = Scale of positive and negative experience; SPT = Subjective probability test; STADI = State-trait anxiety-depression inventory; SWLS = Satisfaction with life scale; TPAS = Types of positive affect scale; Under+Comm = Undergraduate students and community sample; VAS = Visual Analogue Scale; WB = Wellbeing; WEMWBS = Warwick-Edinburgh mental well-being scale; WL = waiting list.

Boselie (2016)a [44] = study 1, Boselie(2016)b [44] = study 2.

Harrist et al. (2007)a [45] = writing conditions, = Harrist et al. (2007)b [45] = talking conditions.

Layous et al. (2013)a [46] = face-to-face conditions, Layous et al. (2013)b [46] = online conditions.

The 26 selected articles (with 29 studies) included 2,909 participants (1,270 in BPS groups, 1,178 in control groups, and 461 in gratitude groups). The majority of them administered the interventions to University students (k = 20), some of them combined University students with the general population (k = 4), and only two studies were carried out completely in the general population (community). Participants’ mean age was 23.56 (range from 17.83 to 35.62), with a standard deviation of 4.53 (range from 1.12 to 13.99), and the mean percentage of women was 74.41% (range from 52.70 to 100). Regarding the components of the intervention, fifteen studies included an imagery component. Specifically, one included explicit instructions for the visualization [49], and fourteen specified a period of time in which participants should visualize their BPS after the writing period (generally, 5 minutes). The majority of the studies (k = 21) gave participants money or University credits as compensation for their participation (vs. no compensation for participating), four studies administered the intervention in small groups (vs. individually), and six through the Internet (vs. face-to-face). With regard to control conditions, only one study used a waiting list as a control group [49], whereas the remaining studies included an active control group that had to write about a neutral topic. Specifically, participants in the control conditions wrote about what they did in the past 24 hours, the past week, or on a typical day [10,16, 26,46,48,50–62], their plans for the coming week or the next day [5,45,63], the layout of a place where they had been earlier [64], early memories [47], a description of a book or a film [65], or a combination of neutral topics [66]. Moreover, seven studies included a gratitude group in addition to the control and BPS groups. Explicitly, four studies asked participants to write lists of things they were grateful for [47,49,53,63], and one study asked participants to write (but not to send) a letter of gratitude to another person who did something for them [61]. In all the included studies, the control and gratitude exercises were equal to the BPS condition in the delivery method, contextual aspects, components, duration, and population (except for the control condition in the study with the waiting list as a control group). The interventions lasted from 1 to 56 days (M = 14), with an intensity ranging from 10 to 75 minutes per week (M = 24), and a magnitude ranging from 20 to 170 minutes of practice in total (M = 45).

Regarding the assessed quality of the studies (see Table 2), scores of the included studies ranged from 4 to 8 on a scale from 0 to 9 (M = 6.58; SD = 1.35). None of the studies met all the quality criteria, and only one study reported concealment of the assessors. All the studies randomized the participants to each condition and used standardized scales. The majority of the studies (k = 28) presented the measures reported in the method section in the results section. Eighteen studies reported baseline comparability between dropouts and completers, and 22 studies reported baseline comparability between BPS and control groups. All studies except one used active control groups. Only half of the studies (15/29) used intention-to-treat analyses, and attrition rates were below 10% in 21 studies.

**Table 2:** Quality assessment per study.

Study	1	2	3	4	5	6	7	8	9	Total
Boehm et al. (2011) [26]	1	1	1	1	0	1	0	0	1	6
Boselie et al. (2014) [60]	1	1	1	1	0	1	1	1	1	8
Boselie et al. (2016)a [44]	1	1	1	1	0	1	1	1	1	8
Boselie et al. (2016)b [44]	1	1	1	1	0	1	1	1	1	8
Boselie et al. (2017) [62]	1	1	1	1	0	1	1	1	1	8
Enrique et al. (2017) [58]	1	1	0	1	0	1	0	1	1	6
Geschwind et al. (2015) [59]	1	1	1	1	0	1	1	1	1	8
Hanssen et al. (2013) [16]	1	1	1	1	0	1	1	1	1	8
Harrist et al. (2007)a [45]	1	0	1	1	0	1	1	1	1	7
Harrist et al. (2007)b [45]	1	0	1	1	0	1	1	1	1	7
King (2001) [5]	1	0	0	1	0	1	1	0	1	5
Layous et al. (2013)a [46]	1	1	1	1	0	1	1	0	1	7
Layous et al., (2013)b [46]	1	1	1	1	0	1	0	0	1	6
Liau et al. (2016) [52]	1	1	0	1	0	1	0	0	1	5
Lyubomirsky et al. (2011) [61]	1	1	0	1	0	1	1	0	1	6
Maddalena et al. (2014) [56]	1	1	0	1	1	1	1	0	1	7
Manthey et al. (2016) [63]	1	1	0	1	0	1	0	0	1	5
Meevissen et al. (2011) [10]	1	1	0	1	0	1	1	0	1	6
Meevissen et al. (2012) [48]	1	1	1	1	0	1	1	1	1	8
Ng (2016) [64]	1	1	1	1	0	1	1	0	1	7
Odou & Vella-Brodrick (2013) [49]	1	1	1	0	0	1	0	1	1	6
Peters et al. (2010) [54]	1	1	1	1	0	1	1	1	1	8
Peters et al. (2013) [55]	1	1	0	1	0	1	1	0	1	6
Peters et al. (2016) [50]	1	0	1	1	0	1	1	1	1	7
Renner et al. (2014) [51]	1	1	1	1	0	1	1	1	1	8
Sheldon et al. (2006) [53]	1	0	0	1	0	1	1	0	0	4
Summerfield (2015) [47]	1	0	0	1	0	1	0	0	1	4
Troop et al. (2013) [65]	1	1	1	1	0	1	1	1	1	8
Yogo et al. (2008) [66]	1	0	0	1	0	1	0	0	1	4
Total	29	22	18	28	1	29	21	15	28	184

Quality criteria: 1 = Randomization, 2 = Baseline comparability (BPS vs. control group), 3 = Baseline comparability (completers vs. dropouts), 4 = Active control group, 5 = Concealment of assessors, 6 = Standardized scales, 7 = Attrition rate ≤ 10%, 8 = Intention-to-treat analyses, 9 = Report bias.

Boselie (2016)a [44] = study 1, Boselie(2016)b [44] = study 2.

Harrist et al. (2007)a [45] = writing conditions, = Harrist et al. (2007)b [45] = talking conditions.

Layous et al. (2013)a [46] = face-to-face conditions, Layous et al. (2013)b [46] = online conditions.

### Mean effect size and heterogeneity

**Fig 2:**
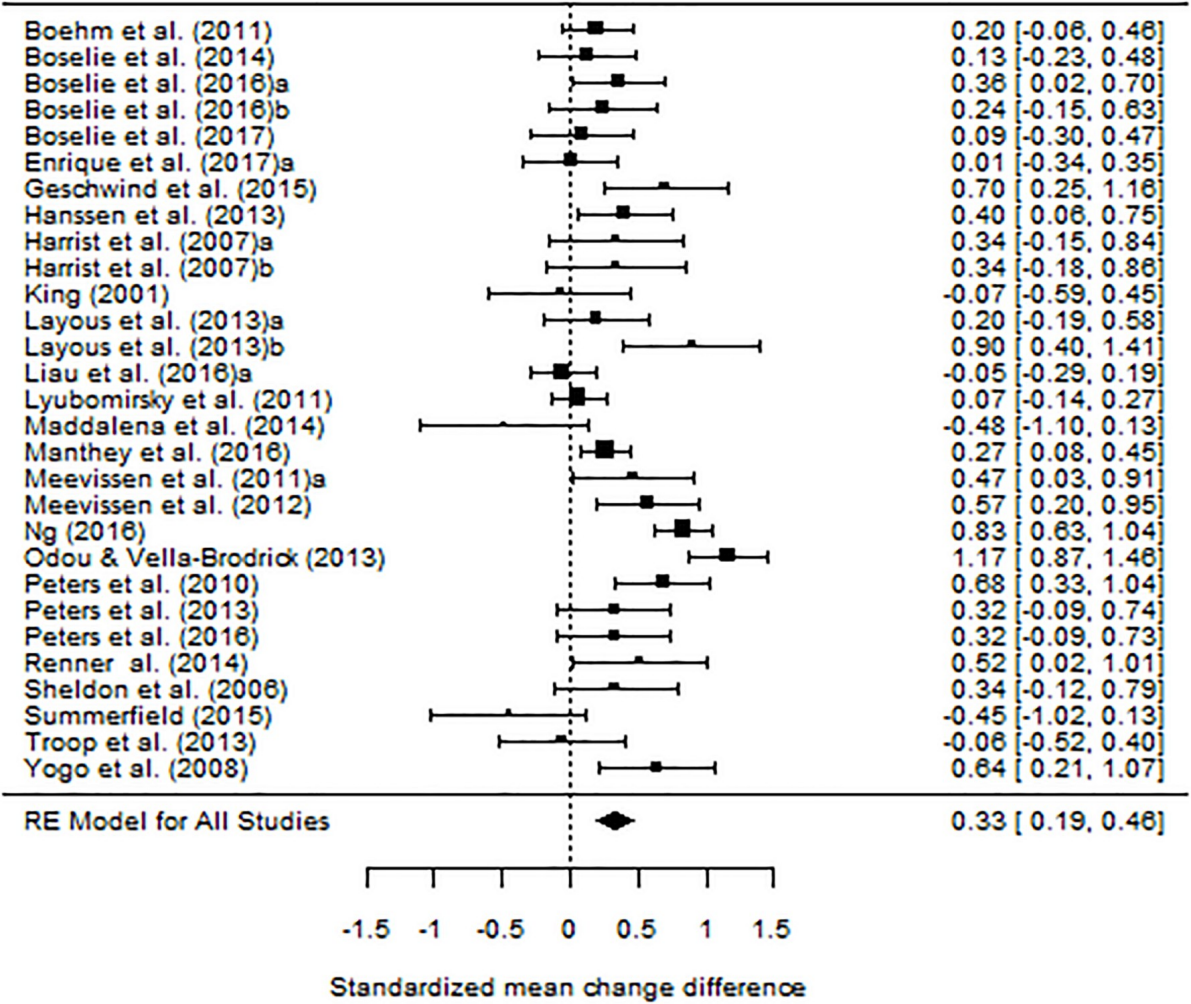
Forest plot displaying the effect sizes (and 95% confidence intervals) for wellbeing.

Table 3 presents the results of the effectiveness of the BPS comparing to control groups for wellbeing, positive affect, negative affect, optimism, and depression. The largest mean effect size was found for positive affect (*d*_+_ = .511), which can be considered a moderate effect size, followed by wellbeing (*d*_+_ = .325) and optimism (*d*_+_ = .334), which reflect medium magnitudes [31]. For negative affect and depression, the obtained effect sizes were considerably small (*d*_+_ = .192 and *d*_+_ = .115, respectively). Fig 2 presents a forest plot for wellbeing effect sizes, and S1 File presents forest plots for positive affect, negative affect, and optimism (Figs A, B and C in S1 File, respectively).

**Table 3:** Mean effect size, 95% confidence intervals, and heterogeneity statistics for the effectiveness of the BPS versus control group.

95% CI						
Outcome measure	*k*	*d*_+_	LL	UL	*Q*	*I*^2^
Wellbeing	29	0.325	0.189	0.461	113.16****	73.83
Positive affect	13	0.511	0.257	0.765	59.78****	79.29
Negative affect	13	0.192	-0.328	0.712	181.89****	94.91
Optimism	13	0.334	0.246	0.422	7.39	0.0
Depression	3	0.115	-0.272	0.502	3.38	42.66

*k* = number of studies. *d*_+_ = mean effect size. LL and UL = lower and upper 95% confidence limits for *d*_+_. *Q* = Cochran’s heterogeneity *Q* statistic; *Q* statistic has *k*– 1 degrees of freedom. *I*^2^ = heterogeneity index.

*p < .05.

**p < .01.

****p < .0001.

Given the similarity of the activities performed in the control groups, which consisted on writing about a neutral topic (see descriptive characteristics of the studies), no further comparisons were performed between the active control groups. However, as mentioned above, all of the included studies compared the BPS to an active control group except for one study that compared the BPS exercise to a non-active control group (waiting list). This study reported effect sizes for wellbeing and positive and negative affect. When the only BPS-non-active control effect size was compared to the BPS-active control effect sizes, statistically significant differences were found for wellbeing, *F*(1, 27) = 9.07, *p* = .006 (BPS-active controls: *d*_+_ = 0.292, 95% CI [0.170, 0.414], *k* = 28; BPS-non-active control: *d* = 1.166, 95% CI [0.583, 1.749]). For positive affect, there were no statistically significant differences, *F*(1, 11) = 0.35, *p* = .565 (BPS-active controls: *d*_+_ = 0.534, 95% CI [0.258, 0.810], *k* = 12; BPS-non-active control: *d* = 0.284, 95% CI [-0.604, 1.171]). Finally, for negative affect, statistically significant differences were found, *F*(1, 11) = 262.12, *p* < .001 (BPS-active controls: *d*_+_ = -0.047, 95% CI [-0.145, 0.050], *k* = 12; BPS-non-active control: *d* = 3.013, 95% CI [2.608, 3.417]). As it can be seen, the average effect sizes for wellbeing and positive affect reported in Table 3 were very similar to those obtained for the BPS-active control comparison. However, for negative affect, the average BPS-active control effect size was practically null.

Table 4 presents the results of the comparison of the effectiveness of the BPS and gratitude interventions for wellbeing, positive affect, and negative affect. The largest mean effect sizes were found for positive affect (*d*_+_ = .326) and negative affect (*d*_+_ = .485), estimates that reflect medium and moderate magnitudes, respectively [31]. For wellbeing, the average effect size was null. Effect sizes presented great heterogeneity, with the *Q* statistics reaching statistical significance and the *I*^2^ indices above 60% in all cases.

**Table 4:** Mean effect size, 95% confidence intervals, and heterogeneity statistics for the effectiveness of the BPS versus gratitude interventions.

95% CI						
Outcome measure	*k*	*d*_+_	LL	UL	*Q*	*I*^2^
Wellbeing	7	0.092	-0.115	0.299	15.609*	63.23
Positive affect	5	0.326	0.011	0.641	17.075**	70.36
Negative affect	5	0.485	-0.301	1.271	65.931****	93.72

*k* = number of studies. *d*_+_ = mean effect size. LL and UL = lower and upper 95% confidence limits for *d*_+_. *Q* = Cochran’s heterogeneity *Q* statistic; *Q* statistic has *k*– 1 degrees of freedom. *I*^2^ = heterogeneity index.

*p < .05.

**p < .01.

****p < .0001.

### Analysis of publication bias

For wellbeing, optimism and positive and negative affect outcomes, publication bias was assessed through Egger tests and funnel plots, applying the trim-and-fill method. In the case of depression, this was not possible due to the small number of studies.

**Fig 3:**
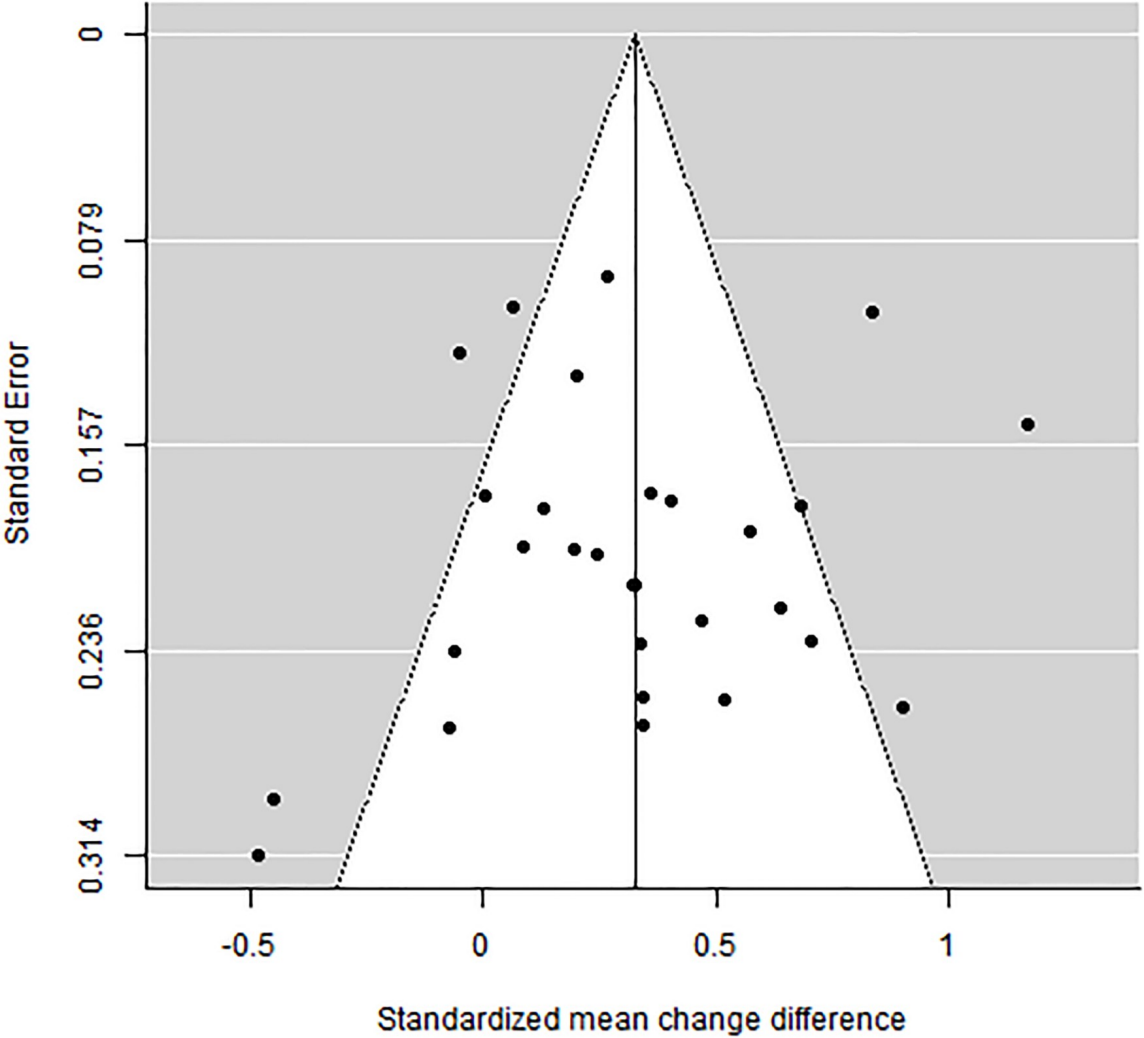
Funnel plot of the 29 standardized mean change difference indices for wellbeing.

For wellbeing, a non-significant result for the interception was obtained with the Egger test: *t*(27) = -1.067; *p* = .296. Fig 3 presents the funnel plot obtained with the original 29 standardized *d* indices. Applying the trim-and-fill method, no standardized mean change differences had to be imputed to achieve symmetry in the funnel plot.

The effect sizes obtained for positive affect, negative affect, and optimism outcomes also exhibited a statistically non-significant result for the intercept (*p* = .206, *p* = .569, *p* = .526, respectively). S1 File presents the funnel plots for the standardized mean change difference indices for each of these outcomes. In particular, for positive and negative affect, the funnel plots were symmetric, and no additional indices had to be imputed (see Figs D and E in S1 File). With regard to optimism, by applying the trim-and-fill method, four additional standardized mean change difference estimates were imputed to the set of the original estimates to achieve symmetry in the funnel plot (see Fig F in S1 File). When a mean effect (and its 95% CI) was calculated with the 13 *d* indices plus the four imputed values, the average effect was *d*_+_ = 0.28 (95% CI [0.19,0.37]). If we compare the new effect to what was obtained with the 13 original *d* indices (*d*_+_ = 0.33; 95% CI [0.26, 0.42]), only slight differences were found. Therefore, the results obtained with the Egger test and the funnel plot obtained using the trim-and-fill method led us to discard publication bias as a threat to these meta-analytic results.

In addition, with the purpose of determining whether publication bias might be a problem of published research on this topic, publication bias methods were also applied by excluding unpublished effect sizes from the analyses. The results for funnel plots, Egger tests, and trim-and-fill method remained unchanged, with the exception of positive affect. In particular, the Egger test reached statistical significance when only published studies were included in the analysis (*p* = .041), leading to evidence of publication bias on this research topic when assessing positive affect.

### Analysis of moderator variables

The results presented in Table 3 about the effectiveness of BPS in comparison with controls show the existence of a large amount of heterogeneity, according to the *Q*_W_ test (*p* < .001). Consequently, the influence of several characteristics related to the intervention, methodology, and participants was examined for wellbeing. Given that positive and negative affect were included in the overall wellbeing outcome, and that only a small number of studies included these constructs, optimism or depression, analyses of moderator variables were not carried out for these outcomes. Table 5 shows the results of the simple meta-regressions applied on continuous moderator variables. All the moderators analyzed revealed statistically non-significant moderating effects with the effect sizes (*p* > .05). However, it is worth noting that the magnitude of the intervention, measured in total minutes of practice, and the mean age (in years) of participants presented marginally statistically significant results, as well as percentages of explained variance above 15% (see Table 5). Specifically, the magnitude of the intervention presented a marginal association with the effect sizes (*p =* .078), with 25% of the variance accounted for, and the mean age showed a marginal association with the effect sizes (*p* = .079), accounting for the 15% of the variance.

**Table 5:** Results of the simple meta-regressions of continuous moderator variables on the effect sizes for wellbeing.

Moderator variable	*k*	*b*_j_	*F*	*p*	*Q*_E_	*R*^2^
Length	29	-0.005	1.775	.194	100.64****	.05
Intensity (minutes per week)	25	-0.007	1.964	.174	50.620***	0
Magnitude (total minutes)	25	-0.003	3.408	.078	44.128**	.25
Mean age (years)	26	0.024	3.351	.079	88.900****	.15
Sex (% female)	29	0.005	0.697	.411	113.051****	0
Methodological quality scale	29	0.044	0.738	.398	107.276****	0
BPS group sample size	29	0.002	0.562	.460	112.39****	0
Control group sample size	29	0.001	0.214	.648	113.157****	0

*k* = number of studies. *b*_j_ = regression coefficient of each predictor. *F* = Knapp-Hartung’s statistic for testing the significance of the predictor (the degrees of freedom for this statistic are 1 for the numerator and *k*– 2 for the denominator). *p* = probability level for the *F* statistic. *Q*_E_ = statistic for testing the model misspecification. *R*^2^ = proportion of variance accounted for by the predictor.

** *p* < .01.

*** *p* < .001.

**** *p* < .0001.

Table 6 presents weighted ANOVAs for the analysis of categorical moderator variables. Of the different moderators analyzed, only the continent where the study was conducted showed a statistically significant result (*p* = .029), accounting for a large percentage of the variance (35%). As it can be seen, the largest mean effect size was yielded by the only study carried out in Oceania (*d*_+_ = 1.166), which was also the only study with a non-active control group, whereas the mean effect sizes for the remaining continents were very similar. In fact, when these analyses were repeated without the Oceania study, this moderator did not reach a statistical association with the effect sizes (*p* = .449).

**Table 6:** Results of the weighted ANOVAs of categorical moderator variables on the effect sizes for wellbeing.

			95% CI		
Moderator variable	*k*	*d*_+_	LL	UL	ANOVA results
Delivery method:					*F*(1,27) = 0.56, *p* = .462 *R*^2^ = 0.0 *Q*_W_(27) = 107.99, *p* < .001
Individually	24	.348	.197	.499	
In groups	5	.217	-.109	.544	
Delivery method:					*F*(1,27) = 0.15, *p* = .701 *R*^2^ = 0.0 *Q*_W_(27) = 113.05, *p* < .001
Online	6	.373	.082	.664	
Face-to-face	23	.311	.154	.468	
Imagery component:					*F*(1,27) = 0.91, *p* = .349 *R*^2^ = 0.0 *Q*_W_(27) = 108.38, *p* < .001
No	14	.260	.065	.456	
Yes	15	.387	.197	.577	
Compensation for participation:					*F*(1,27) = 0.23, *p* = .635 *R*^2^ = 0.0 *Q*_W_(27) = 113.14, *p* < .001
No	8	.375	.121	.628	
Yes	21	.304	.139	.469	
Target population:					*F*(2,26) = 1.61, *p* = .220 *R*^2^ = .05 *Q*_W_(26) = 102.22, *p* < .001
Community	2	.671	.207	1.135	
Undergraduate	23	.318	.167	.469	
Under+Comm	4	.164	-.192	.521	
Continent:					*F*(3,25) = 3.53, *p* = .029 *R*^2^ = .35 *Q*_W_(25) = 71.45, *p* < .001
Europe	16	.297	.134	.460	
N. America	9	.208	-.018	.434	
Oceania*	1	1.166	.576	1.755	
Asia	3	.462	.123	.801	

*k* = number of studies. *d*_+_ = mean effect size. LL and UL = lower and upper 95% confidence limits for *d*_+_. *F* = Knapp-Hartung’s statistic for testing the significance of the moderator variable. *Q*_W_ = statistic for testing the model misspecification. *R*^2^ = proportion of variance accounted for by the moderator. Under+Comm = Undergraduate students and community sample.

*The largest mean effect size was obtained in the only study carried out in Oceania [49]. When this study was extracted from the calculus, no significant results emerged.

## Discussion

This is the first meta-analysis to examine the effectiveness of the Best Possible Self intervention, compared to controls, on wellbeing and other related outcomes. It included 26 articles (with 29 studies) and a total of 2,909 participants. Medium to moderate effect sizes were found for wellbeing, optimism, and positive affect, whereas the effects sizes found for negative affect and depressive symptoms were considerably small [31,67]. The effect sizes obtained for wellbeing (*d*_+_ = 0.325) in this work are lower than the effect sizes found in the meta-analyses of PPIs conducted by Sin and Lyubomirsky [2] (*d* = 0.61), but more similar that the ones found in the meta-analysis conducted by Bolier and colleagues [3], being greater in the case of psychological wellbeing (*d* = .20) and slightly smaller (but almost equal) in the case of subjective wellbeing (*d* = .34). These meta-analyses showed that PPIs (regardless of the specific type of PPI) produced medium to moderate effects on wellbeing [31], and similar results were found in this work on the effectiveness of the BPS intervention.

Moderator analyses of the quantitative variables did not show any significant moderating effects on wellbeing outcomes. However, in light of the large number of studies included, the marginal effects observed in these analyses are worth mentioning. Regarding the magnitude of the intervention, the negative slope suggests that interventions that included fewer total minutes of practice produced larger effect sizes. These results might indicate that processes such as the hedonic adaptation could affect the effectiveness of interventions practiced for longer periods of time, causing the effects of shorter practices to fade when participants are asked to practice more time [68]. In addition, the positive slopes for age showed that the interventions carried out with older participants were associated with the largest effect sizes. Nevertheless, these effects should be understood within a cohort of young adults from 18 to 35 years, indicating that interventions carried out with older participants in this age range lead to better outcomes. In addition, although no significant results emerged regarding the target population, larger effect sizes were observed in the community samples in comparison with the undergraduate students (usually, younger than the community samples). These results somehow contradict the theoretical assumptions of Lyubomirsky and Layous [23], who hypothesized that PPIs with a future-time orientation, like the BPS intervention, would be more beneficial for young people. It is possible that younger participants might find it difficult to envision their best possible self as their future is still undefined (e.g. which will be one’s future occupation or whether one will raise a family), while older participants might be more connected with their values and may have more established life goals due to their life experiences and normative factors. In any case, these results should be interpreted in the context of studies with considerably young participants and with a limited age range. Further research is needed with older samples in order to explore the role of age in this intervention, as well as with more heterogeneous samples (with both young and older participants). With regard to the moderator analyses of the categorical variables, none of them showed a significant moderating effect on wellbeing.

Overall, the moderator analyses observed in this study support statements from a recent qualitative review of the BPS intervention suggesting that BPS is a flexible and effective intervention, regardless of the delivery method or the participants’ characteristics [6].

The BPS exercise has been widely used to specifically promote optimism. Interestingly, the effect size of the BPS intervention on optimism is similar to the one obtained for wellbeing, which suggests that its effectiveness is similar for both constructs. Overall, the effect sizes obtained for optimism outcomes in our meta-analysis are lower than those observed in the meta-analysis by Malouff and colleagues [7]. In this case, the different studies included and the type of calculation of the effect size could account for this difference.

Regarding depression, only three studies could be entered into the effect size calculus, which was small (*d*_+_ = 0.115). These results are slightly lower than those presented in the last meta-analysis of PPIs [3] (*d* = .23), although both are considered small [4,31]. The review by Loveday [6] concluded that BPS can be used with depressive patients, among others. Nevertheless, considering the small number of included studies that assessed depressive symptoms, quantitative results for the effects of BPS interventions on depression should be viewed with caution.

Because a large number of studies included the PANAS scale [11], we were able to conduct a separate meta-analysis for the effects on positive and negative affect assessed with this specific questionnaire. This is one of the most widely used scales to measure positive and negative mood, and it has been validated in many countries, showing good psychometric properties in numerous studies [69–71]. Effects of BPS on positive affect showed a moderate effect size of *d*_+_ = .511, which was larger than the effect sizes obtained for the other related outcomes. By contrast, a small effect size was found for negative affect (*d*_+_ = .192) and excluding the only study with a non-active control group, this effect size was null (*d*_+_ = -0.047). These results imply that the BPS exercise might be more effective in increasing positive affect than in decreasing negative affect, which is consistent with the PPIs’ aim of promoting positive emotions rather than decreasing negative emotions.

The fact that some studies included a gratitude intervention group in addition to BPS and controls made it possible to conduct a specific meta-analysis on the effectiveness of the BPS compared to gratitude interventions. A medium effect size was found for positive affect (*d*_+_ = .326), and a moderate effect size was found for negative affect (*d*_+_ = .485) [31]. The effect size on wellbeing was quite small (*d*_+_ = .092). It is possible to infer that the BPS seems to produce better results for positive and negative affect than gratitude interventions. However, a small number of studies were included in the analyses, and more research is needed to extend the knowledge about the comparability of these two PPIs.

No indication of publication bias was found in this meta-analysis for any of the different outcomes assessed. It included grey literature, which, along with some studies with negative results, might have helped to overcome the absence of trimmed studies by providing a more complete picture of the field. When considering the published research on this topic (thus excluding the unpublished works included in this meta-analysis), evidence of publication bias was only found for positive affect, and it did not appear in any of the remaining variables, which agrees with a recent meta-analysis on psychological wellbeing conducted by Weiss and colleagues [72].

This study has some limitations. First, regarding the quality of the included studies, none of them met all the quality criteria. For example, only one study included the concealment of the assessors, half of the studies did not use intention-to-treat analyses, and 11 of the 29 studies did not analyze baseline comparability between completers and dropouts (considering that some of the remaining 18 studies did not have any dropouts). Second, the type of population included in the studies was mainly based on University students and young participants, which limits the generalizability of the findings. This is a common issue in Psychology research [73,74], and future studies need to consider broadening the population in which the studies are carried out. Along the same lines, none of the studies (not even the ones that measured depression) delivered the intervention to clinical patients. Hence, it is still necessary to study the effectiveness of the BPS in this population. Third, regarding quantitative analyses, we were not able to adjust a multiple meta-regression model that included a subset of characteristics of studies that could explain the variability in the effect sizes on wellbeing. In addition, the analyses of the differences on the effect sizes between the studies with active or non-active control conditions included only one study in the non-active control group, which limits the strength of the analyses as a result of the lack of variance in this group. Fourth, follow-up analyses were not included due to the small number of studies that reported them: only three studies included follow-up measures beyond three months [26,58,63], which impeded the exploration of long-term effects. Future studies should include follow-ups in order to explore the maintenance of the results in the long-term and the ways to overcome potential obstacles, such as hedonic adaptation to the benefits produced by these interventions [68]. Finally, our approach of averaging dependent effect sizes from the same study could be considered a suboptimal strategy, as it might be more appropriate to apply methods to statistically integrate dependent effect sizes, such as the robust variance estimation method [75] or multilevel meta-analysis [76].

The results of this meta-analysis have several implications for research and clinical practice. Notably, the BPS has been shown to be an effective intervention to improve positive affect, wellbeing, and optimism. Small effect sizes were obtained for negative affect and depressive symptoms. These results indicate that this intervention is more effective in increasing positive outcomes than in decreasing negative ones, and this is consistent with the framework of PPIs, which were conceived to cultivate positive emotions [2]. For this reason, it is possible to state that the BPS exercise is able to produce the desired effects of these type of exercises and, therefore, can be an advantageous strategy to increase participants’ wellbeing.

In relation to the moderator variables, analyses showed that the intervention can be equally effective independently of the delivery method, contextual aspects, and components of the intervention: whether administered individually or in groups, online or face-to-face, with or without an explicit imagery component, similar outcomes seem to be produced. Marginally significant differences were found in the characteristics of the population where the intervention was administered, specifically regarding age, indicating that the age of the participants could play a role in the effectiveness of the intervention. It is important for future studies to include more heterogeneous age groups and older participants in order to address this issue. As to the duration of the intervention, no differences were found in length and intensity, but a marginally significant difference emerged in the magnitude of the intervention. This result suggests that shorter practices (in total number of minutes) may lead to more benefits from the BPS. However, these results should be further explored. In this regard, further studies that include qualitative data (for example, content analyses of the texts) could help to shed light on these results, and on possible variables that might play a role in the effectiveness of the BPS which cannot be addressed through a quantitative approach.

In conclusion, this study contributes to a better understanding of the effectiveness of a widely used PPI. Psychologists and other professionals can consider administering the BPS intervention if they are interested in increasing their clients’ wellbeing levels, given that the BPS emerged as a valuable intervention to increase wellbeing, optimism, and positive affect.

## Supporting information

S1 Checklist PRISMA 2009 Checklist. (PDF)

S1 File Forest and funnel plots. Forest plots displaying the effect sizes for positive affect, negative affect and optimism; and funnel plots for positive affect, negative affect and optimism.
(PDF)
