# Supplementary material for: Effects of the Best Possible Self intervention: A systematic review and meta-analysis
Source: PLoS One. 2019 Sep 23;14(9):e0222386. doi: 10.1371/journal.pone.0222386 (PMC6756746; doi:10.1371/journal.pone.0222386)

SUPPLEMENTARY FILE 1

Fig A. Forest plot displaying the effect sizes (and 95% confidence intervals) for positive affect.

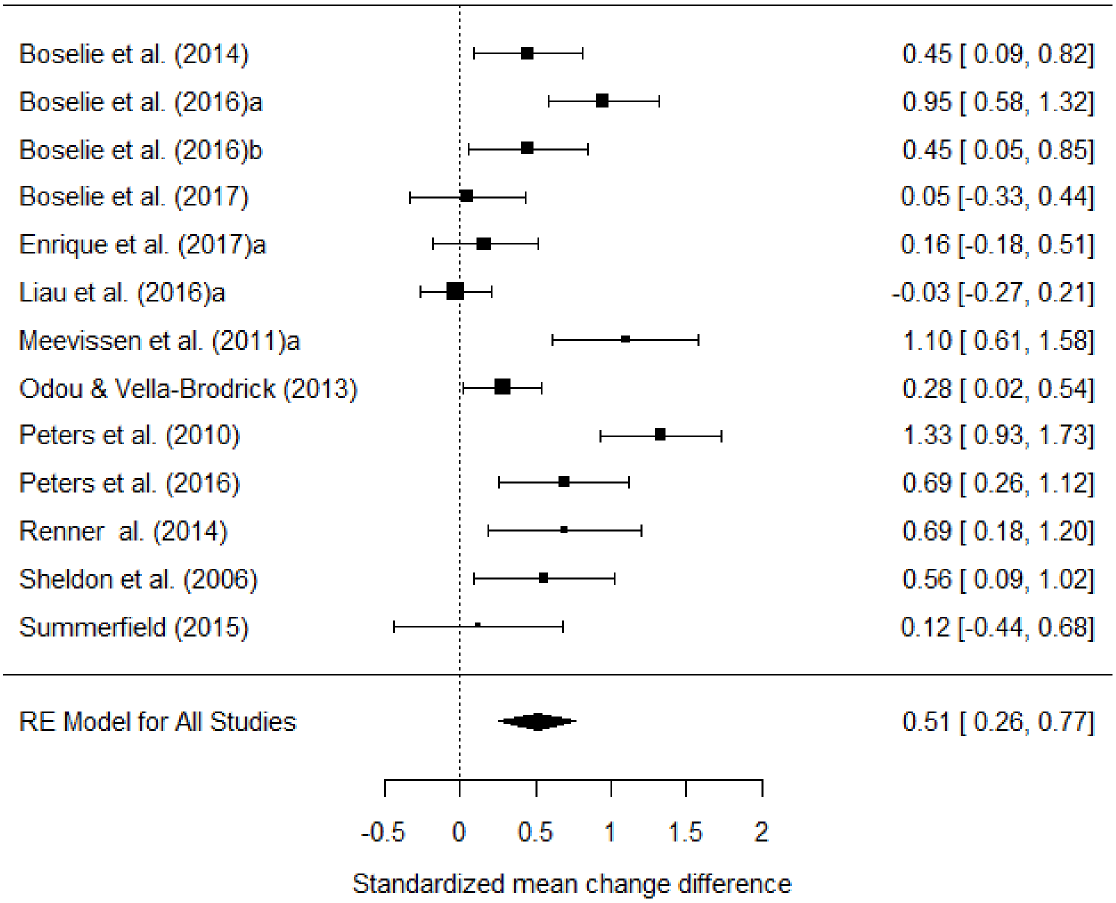

Fig B. Forest plot displaying the effect sizes (and 95% confidence intervals) for negative affect.

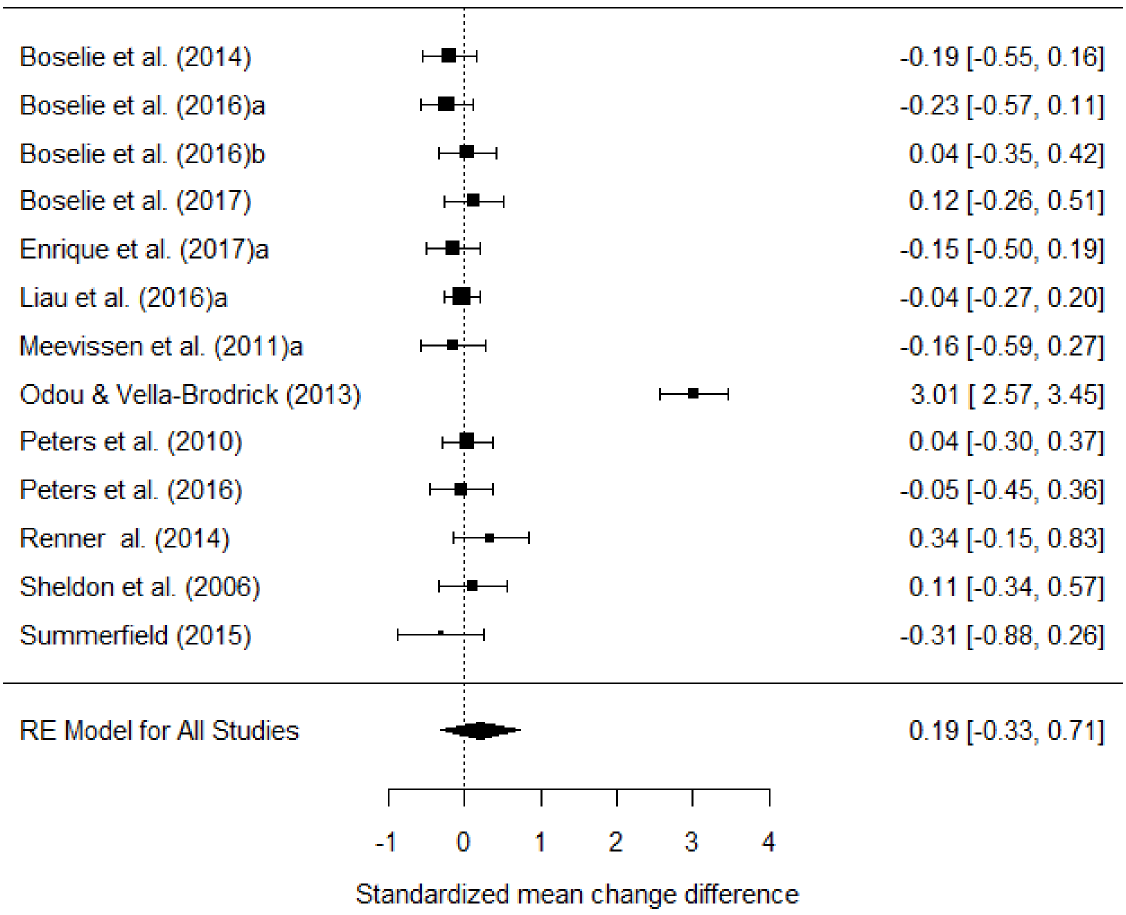

Fig C. Forest plot displaying the effect sizes (and 95% confidence intervals) for optimism.

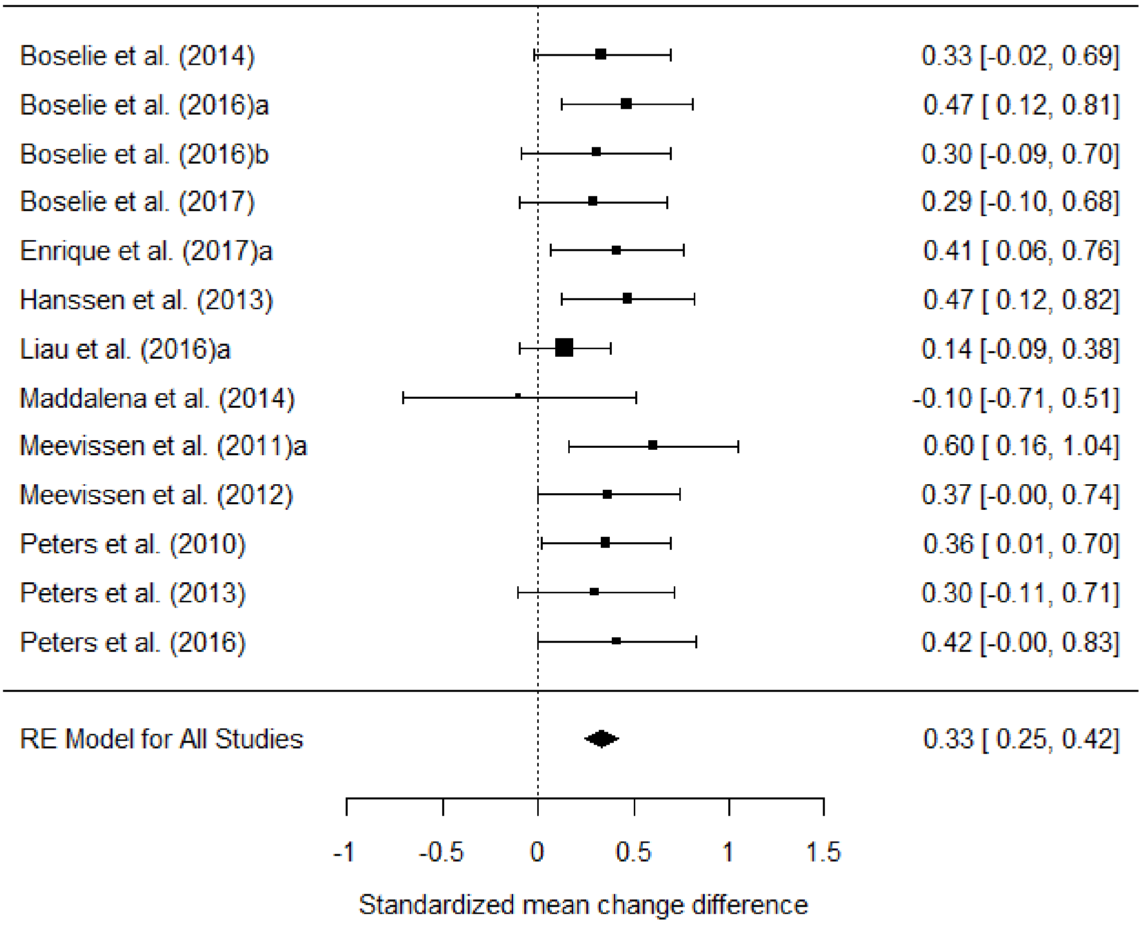

Fig D. Funnel plot of the 13 standardized mean change difference indices for positive affect.

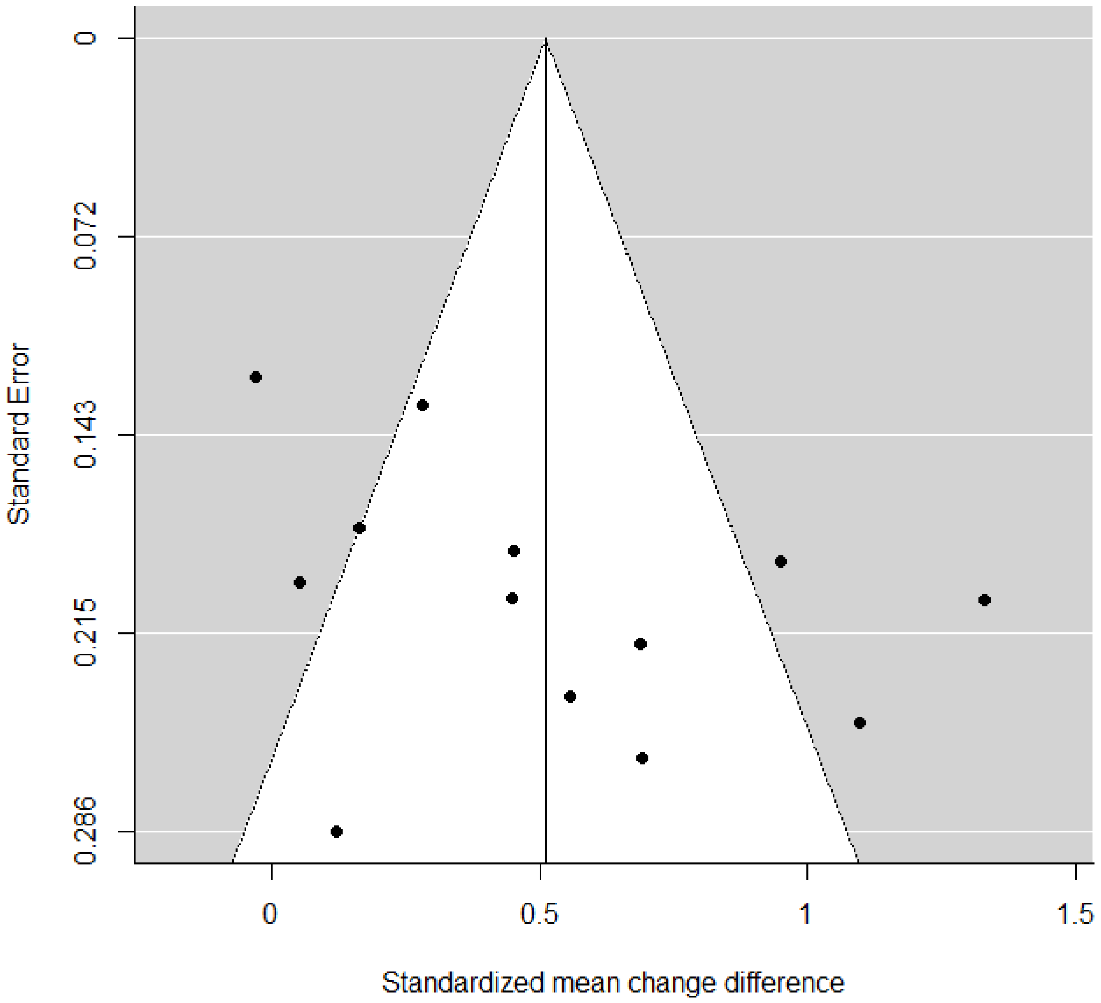

Fig E. Funnel plot of the 13 standardized mean change difference indices for negative affect.

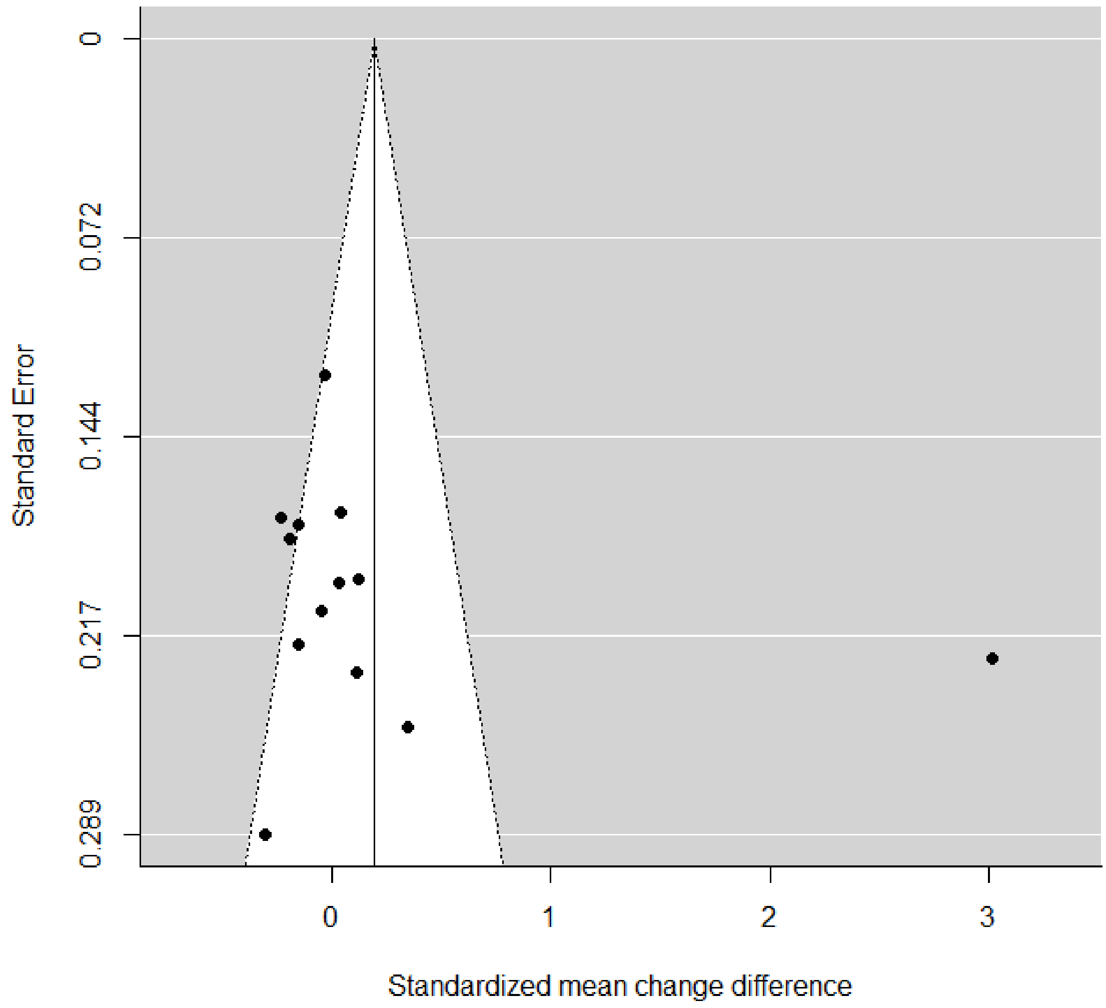

Fig F. Funnel plot of the 13 standardized mean change difference indices for optimism. The four white circles are imputed effect sizes by means of the Duval and Tweedie´s trim-and-fill method.

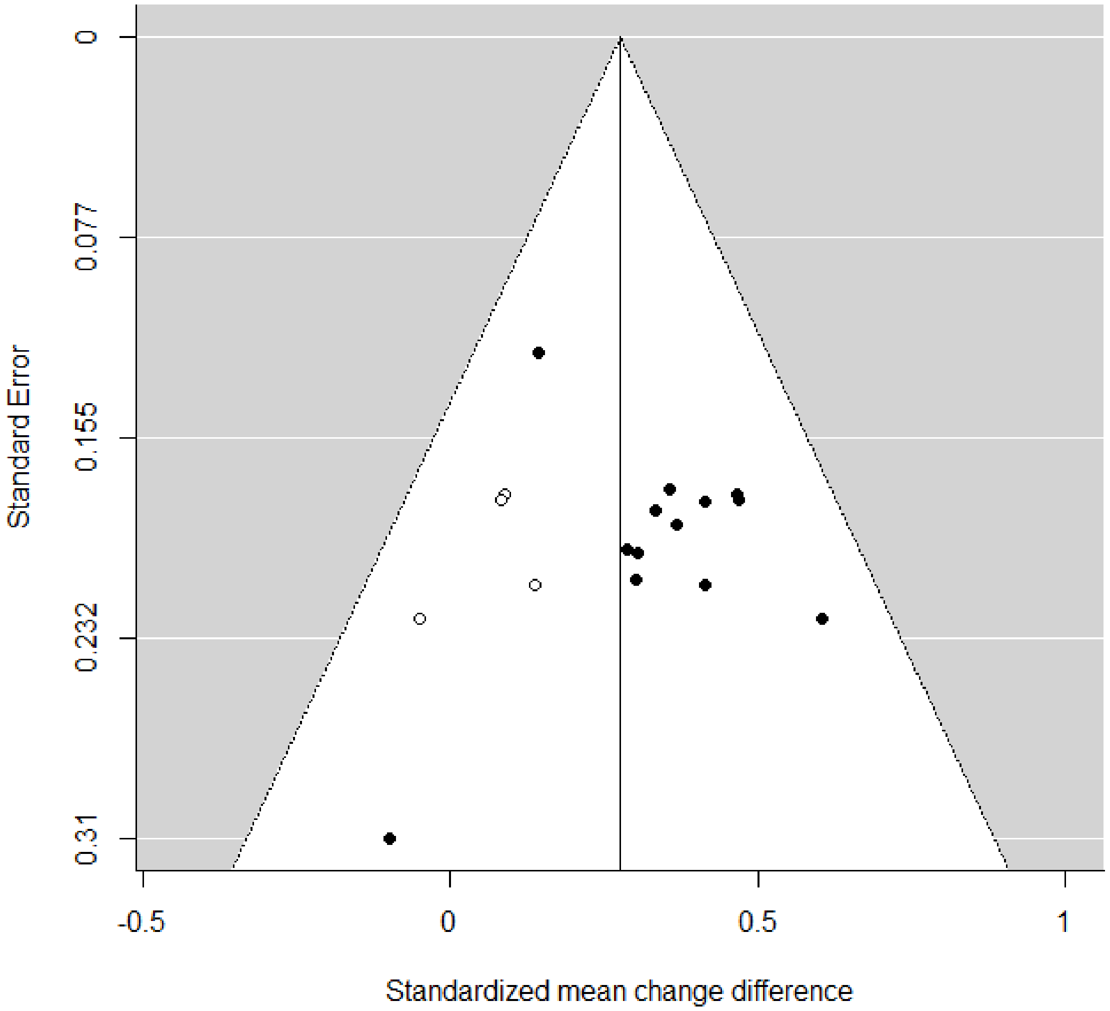

Supplement: S1 File — Forest plots displaying the effect sizes for positive affect, negative affect and optimism; and funnel plots for positive affect, negative affect and optimism. (PDF) [file pone.0222386.s002.pdf]
